# Characteristics and prognosis of primary pulmonary osteosarcoma: a pooled analysis

**DOI:** 10.1186/s13019-022-02010-6

**Published:** 2022-09-29

**Authors:** Weijia Huang, Han-Yu Deng, Deyan Li, Peiwei Li, Kai Xu, Yu-Xiao Zhang, Jia-Hui Weng, Qinghua Zhou

**Affiliations:** 1 Lung Cancer Center, West China Hospital, Sichuan University, No. 37 Guoxue Alley, Chengdu, 610041 People’s Republic of China; 2 West China School of Medicine, Sichuan University, Chengdu, People’s Republic of China; 3 Operation Room, West China Hospital, Sichuan University, Chengdu, People’s Republic of China

**Keywords:** Primary osteosarcoma, Lung, Prognosis, Predictors, Nomograms

## Abstract

**Background:**

Primary pulmonary osteosarcoma (PPOS) is an uncommon malignancy originating from the lung with low incidence, and its clinical characteristics and prognosis have not been systematically reported. Therefore, we aimed to recognize the prognostic factors and constructed a survival prediction model for PPOS.

**Methods:**

We collected the data from the Surveillance, Epidemiology, and End Results database and systematic review of previous studies. Demographical and clinical characteristics, radiographic manifestations, treatment modalities, and prognosis were analyzed. A prediction model via nomogram was constructed and then evaluated by the concordance index (C-index) and the receiver operating characteristic (ROC) curve.

**Results:**

A total of 49 cases were included for analysis with a median age of 67 years old (range 33–94 years), of which 32 (65.3%) were male. The median size was 6 cm (range 1.8-25 cm), and the median overall survival (OS) was eight months (interquartile range 4.5–12 months) with a 1-year OS rate of 30.8%. Tumor size over 7 cm (hazard ratio [HR] = 2.98; *P* = 0.018) and those without microscopic findings of osteoid found in the tumors (HR = 2.11; *P* = 0.048) were referred to a poor OS, while surgery was associated with an improved OS (HR = 0.20; *P* < 0.001). The C-index of the nomogram prediction model was 0.771, and the area under curve, sensitivity and specificity of the ROC curve were 0.818, 0.848 and 0.800, respectively.

**Conclusions:**

Patients with PPOS had a poor prognosis, and tumor size was mostly prognostic. Surgery seemed to be an effective treatment, and the prediction model with a nomogram in our study could effectively predict the prognosis of patients with PPOS.

**Supplementary Information:**

The online version contains supplementary material available at 10.1186/s13019-022-02010-6.

## Introduction

Primary pulmonary osteosarcoma (PPOS) is a rare neoplasm accounting for only 0.01% of malignancies that originated primarily from the lung [1, 2]. In contrast to osteosarcoma originating from the skeletal system and is common in young patients, PPOS tended to suffer among the patients over 50 years [2–5] and was characterized with osteoid formation [6, 7]. To our knowledge, PPOS was first reported by Edward in 1933 [8], and patients with PPOS were found to have a poor prognosis, with a median overall survival (OS) of 10.0 months [4]. In most cases, the tumor might grow to large size of over 5 cm before diagnosis, and PPOS would have a rapid progression after diagnosis [5, 9–11]. The preferred management for PPOS remains controversial, while extensive and radical surgical resection might lead to favored survival [2, 5, 12]. Therefore, the role of radiotherapy or chemotherapy has not been well studied among patients with PPOS [5, 13].

Due to its extremely low incidence, the clinical characteristics and prognostic factors of PPOS have not been systematically demonstrated, and a preferred summary of rare diseases could be achieved based on the Surveillance, Epidemiology, and End Results (SEER) database [14]. Therefore, in this study, we analyzed the documented cases from the SEER database and systematically reviewed the reported cases of PPOS to reveal the clinical characteristics and prognosis. By pooling all these available cases together, we aimed to investigate the potential effective treatment and construct a prognosis prediction model to provide evidence for the clinical practice of PPOS.

## Patients and methods

### Patient selection

SEER* Stat (version 8.3.6) was employed for the first cohort, acquired in May 2020 at the SEER website (http://www.seer.cancer.gov). This study was conducted in accordance with the amended Declaration of Helsinki, and the ethics approval of the research was not required by the Ethics Committee on Biomedical Research, West China Hospital of Sichuan University, due to the anonymous data obtained from the SEER database. Patients diagnosed with PPOS between 1975 and 2016 were included (International Classification of Diseases-10 site code: C34.0-C34.3, C34.8-C34.9; morphology code: 9180-9187, 9192-9194). As for the second cohort, we included the reported cases from a systematic literature review, for which two investigators conducted in the Medline database independently with the following searching terms: ‘primary’ AND ‘lung OR pulmonary’ AND ‘osteogenic sarcoma OR osteosarcoma’. Studies were collected on April 15, 2020, and only studies published in English were included. The cases from case reports, case series, and retrospective studies were included, while those only presenting images without describing the case in detail were excluded.

### Data extraction and variables

We first checked for potential overlapping cases between the first and second cohorts via simultaneous identification of the year of diagnosis and baseline characteristics, and no records were duplicated. Thus, we pooled all these patients together as a study cohort for final analysis, and the discrepancy was reviewed and determined by a third investigator. As for the first cohort, demographical and clinical characteristics, treatment, and survival were recorded. Apart from the variables mentioned above, clinical manifestations, radiographic manifestations, methods for definitive diagnosis, and microscopic findings were also collected for the second cohort. As for the data curation, age was subdivided into two groups (< 65 years and ≥ 65 years), and tumor size was stratified into two groups with a cut-off value of 7 cm, which showed favoured discrimination via receiver operating characteristic (ROC) curve method. The treatment modalities included surgical resection, chemotherapy, and radiotherapy, and the sequence of treatment and completeness of surgical resection were not otherwise specified. Survival analysis was conducted for OS and cancer-specific survival (CSS), in which the survival time was calculated from diagnosis to death or last follow-up in most cases and from onset of the disease to death for those diagnosed by autopsy. Only patients with a definite survival time were included in the survival analysis.

### Statistical analysis

The difference between groups was compared by the Chi-square test for categorical variables and by Student’s T-test or Fisher’s exact test for quantitative variables. Kaplan–Meier analysis was employed for univariable analysis. The Cox proportional hazard model was used to identify the independent prognostic factors in multivariable analysis, in which we used a backward elimination method to select variables with statistical difference [15], and the variables with clinical implications were also included. A *P *value < 0.05 was deemed statistically significant.

The nomogram was employed to visualize the multivariable analysis, in which the total points of each prognostic factor would be summed and correlated linearly to a possibility of 1-year and 2-year OS rate [16]. The concordance index (C-index) and ROC curve were conducted to validate the accuracy of the prediction model. The C-index was devoted to assessing the model’s prediction ability, and it referred to a favoured prediction ability when it came to one and poor ability when it comes to 0.5 [17]. The area under the curve (AUC) and the value of sensitivity and specificity of the ROC curve would also help to show the prediction ability. The statistical analyses were conducted by R 3.6.1(The R Foundation for Statistical Computing, Vienna, Austria).

## Results

### Baseline characteristics

A total of 49 cases were included in this study, in which 11 cases were included as the first cohort from the SEER database, and 38 cases as the second cohort from a systematic literature review of 29 studies [1–3, 5, 6, 8–13, 18–35]. There was no overlapping patient between the two cohorts. The baseline characteristics of patients in the first and second cohorts were summarized in detail in Additional file 1: Tables 1–3. The median age at diagnosis was 67 years (range 33–94 years), and 32 (65.3%) were male (Table 1). The median tumor size was 6 cm (range 1.8–25 cm), and 38.8% (n = 19) of the tumors were larger than 7 cm. About one-third (n = 18, 36.7%) were located in the peripheral part of the lung, and distant metastases were found in 20.4% (n = 10).

**Table 1:** Demographics and characteristics of tumor in all patients with primary pulmonary osteosarcoma

Characteristics	Number	%
Age		
< 65	20	40.8
≥ 65	29	59.2
Sex		
Male	32	65.3
Female	17	34.7
Laterality		
Left	26	53.1
Right	22	44.9
NA	1	2.0
Location		
Lower lobe	17	34.7
Middle lobe	4	8.2
Upper lobe	17	34.7
NA	11	22.4
Location		
Central	12	24.5
Peripheral	18	36.7
NA	19	38.8
Size (cm)		
≤ 7	25	51.0
> 7	19	38.8
NA	5	10.2
Metastasis		
Metastasis	10	20.4
No/NA	39	79.6
Year of diagnosis		
< 2002	23	46.9
≥ 2002	26	53.1
Surgery		
Yes	32	65.3
None	17	34.7
Chemotherapy		
Yes	10	20.4
None	39	79.6
Radiotherapy		
Yes	11	22.4
None	38	77.6

*PPOS* primary pulmonary osteosarcoma, *NA* not available

As for clinical manifestations, nine (18.4%) cases were asymptomatic at diagnosis, and twelve (24.5%), four (8.2%), eight (16.3%), and ten (20.4%) cases were found to have chest pain, fever, cough, and dyspnea before diagnosis, respectively. Pleural effusion was found in nine (18.4%) cases, calcification in 16 (32.7%) cases, and intensive uptake on bone scintigraphy in nine (18.4%) cases. In total, around one-third (n = 16, 32.7%) of the cases achieved a definite diagnosis from pathological examinations of surgical specimens. Osteocyte, osteoid, and chondrocyte (including chondroblast and chondroid) were found in 22 (44.9%), 24 (49.0%) and 17 (34.7%) of the cases in the microscopic pathological examination, respectively.

### Treatment and prognosis

**Fig. 1:**
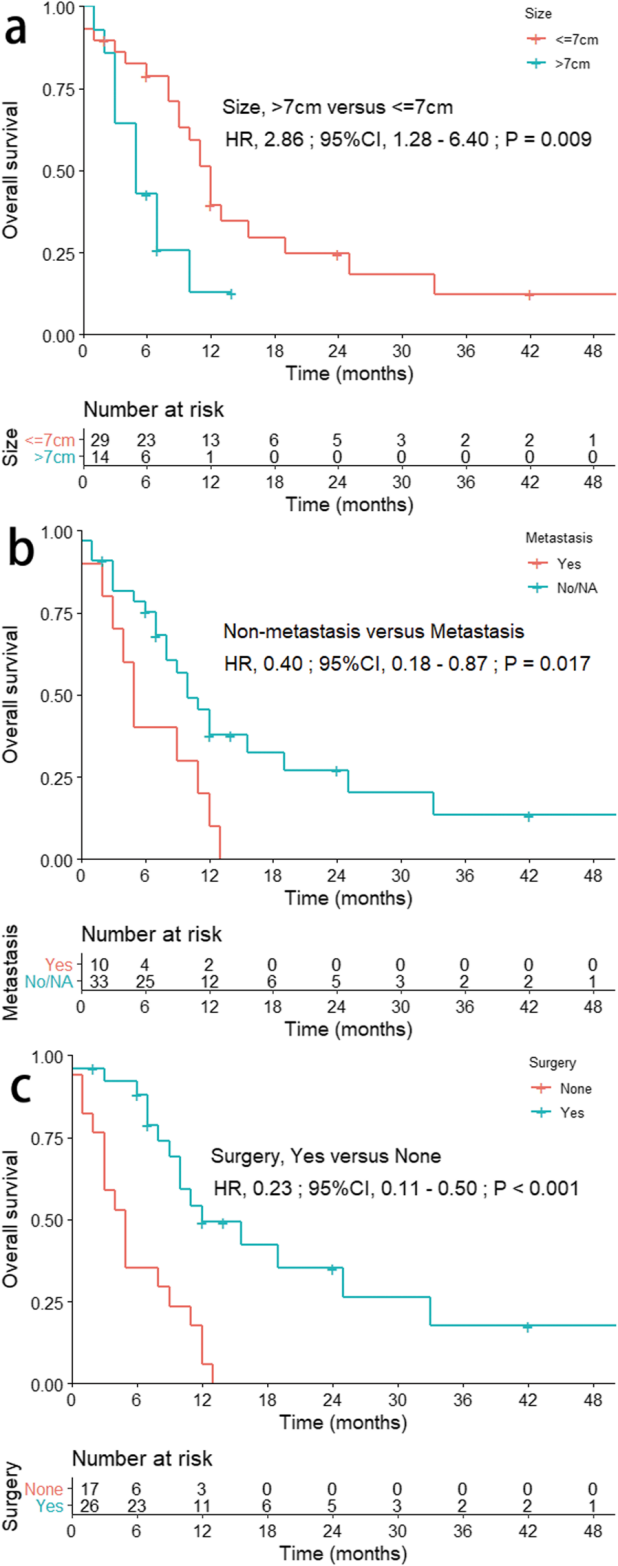
Overall survival in patients with primary pulmonary osteosarcoma stratified by tumor size (**a**; *P* = 0.009), metastasis (**b**; *P* = 0.017), and surgical performance (**c**; *P* < 0.001). Abbreviations: HR, hazard ratio; CI, confidential interval; No/NA, none or not applicable

In total, there were 32 patients (65.3%) recorded to have surgery, 10 patients (20.4%) to receive chemotherapy, and 11 patients (22.4%) to receive radiotherapy (Table 1, Additional file 1: Table 1, and Additional file 1: Table 3). While six cases were excluded in further survival analysis due to the unavailable survival in the raw data, and thus, 43 patients were included in subsequent survival analysis. The median OS of these patients was eight months (interquartile range 4.5–12 months), and the 1-year OS and CSS rates were 30.8% and 38.5%, respectively. In the univariable analysis, surgery was associated with improved survival (median OS, ten versus five months; OS, HR = 0.23, 95% CI 0.11–0.50; *P* < 0.001; Fig. 1). Tumor size (> 7 cm versus ≤ 7 cm, HR = 2.86; 95% CI 1.28–6.40; *P* = 0.009), metastatic diseases (non-metastasis versus metastasis, HR = 0.40; 95% CI 0.18–0.87; *P* = 0.017), and pleural effusion (absence versus presence, HR = 0.35; 95% CI 0.15–0.83; *P* = 0.016) were also significantly associated with OS (Fig. 1; Table 2).

**Table 2:** Univariable analysis of overall survival and cancer-specific survival in all patients and patients without metastasis

Characteristics	All patients^a^		Patients without metastasis^a^	
	OS *P* value	CSS *P* value	OS *P* value	CSS *P* value
Demographics				
Age	0.099	0.188	0.316	0.656
Sex	0.432	0.442	0.818	0.805
Tumor characteristics				
Laterality^b^	0.324	0.384	0.194	0.260
Location (UL, ML or LL)^c^	0.406	0.728	0.219	0.445
Location (central or peripheral)^d^	0.433	0.664	0.368	0.539
Size^e^	0.009	0.023	0.043	0.108
Recorded in situ tumor(s)^f^	0.572	0.352	0.688	0.463
Metastasis	0.017	0.002	–	–
Imaging manifestations				
Pleural effusion on X-ray/CT	0.016	0.017	0.014	0.025
Calcification found in pulmonary mass on CT	0.048	0.034	0.033	0.012
Intensive uptake on bone scintigraphy	0.999	0.926	0.886	0.701
Microscopic findings				
Osteocyte	0.739	0.884	0.240	0.234
Osteoid	0.152	0.491	0.278	0.915
Chondrocyte^g^	0.922	0.910	0.886	0.929
Treatment				
Surgery	< 0.001	< 0.001	< 0.001	< 0.001
Chemotherapy	0.343	0.682	0.601	0.859
Radiotherapy	0.642	0.896	0.266	0.596

*OS* overall survival, *CSS* cancer-specific survival, *UL* upper lobe, *ML* middle lobe, *LL* lower lobe, *CT* computed tomography

^a^Patients with unknown survival status or time were excluded for analysis

^b^The cases with uncertain laterality were classified as the left lung

^c^The cases with an uncertain lobe location were classified as the upper lobe of the lung

^d^The cases with uncertain location (central or peripheral) were classified as a peripheral site of the lung

^e^The cases with an uncertain tumor size were classified as lower than 7 cm

^f^The cases with an uncertain number with recorded in situ tumor(s) were classified as single in situ tumor

^g^Chondrocyte was referred to as chondrocyte, chondroblast, or chondroid

In the multivariable analysis, surgical performance, tumor size, and microscopic findings of osteoid were identified as the independent prognostic factors (Table 3). Patients who received surgery had a significantly favoured OS (HR = 0.20; 95% CI 0.09–0.45; *P* < 0.001) and CSS (HR = 0.14; 95% CI 0.05–0.36; *P* < 0.001), and tumor over 7 cm was referred to an impaired OS (HR = 2.98; 95% CI 1.21–7.34; *P* = 0.018). In addition, patients without microscopic findings of osteoid were also found to have a significantly poor OS (HR = 2.11; 95% CI 1.01–4.42; *P* = 0.048).

**Table 3:** Multivariable analysis of overall survival and cancer-specific survival in all patients^a^

Characteristics	OS		CSS	
	HR (95% CI)	*P* value	HR (95% CI)	*P* value
Size (cm)				
≤ 7	Ref		Ref	
> 7	2.98 (1.21–7.34)	0.018	3.08 (1.09–8.67)	0.033
Microscopic findings of osteoid				
Found	Ref		Ref	
No/NA	2.11 (1.01–4.42)	0.048	1.68 (0.74–3.79)	0.215
Surgery				
None	Ref		Ref	
Yes	0.20 (0.09–0.45)	< 0.001	0.14 (0.05–0.36)	< 0.001
Chemotherapy				
None	Ref		Ref	
Yes	0.57 (0.21–1.56)	0.276	0.74 (0.26–2.08)	0.571

*OS* overall survival, *CSS* cancer-specific survival, *HR* hazard ratio, *CI* confidence interval, *Ref.* reference, *NA* not available

^a^Patients with unknown survival status or time were excluded for analysis

### Construction of the nomogram

**Fig. 2:**
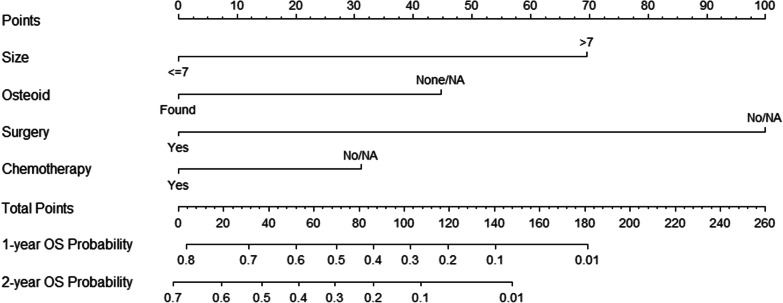
A nomogram to predict the overall survival in patients with primary pulmonary osteosarcoma one and two years after diagnosis. The concordance index was 0.771 (95% CI 0.691–0.851). Abbreviations: Osteoid, microscopic findings of osteoid tissue in the tumor; OS, overall survival; NA, not available

**Fig. 3:**
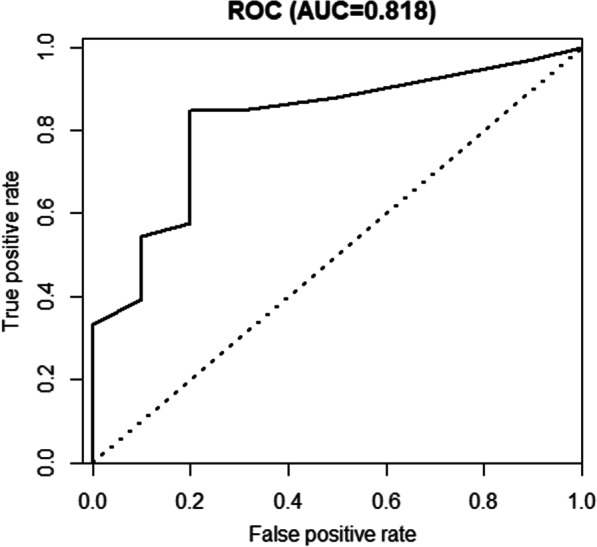
Receiver operating characteristic (ROC) curve to validate the prediction model of overall survival in patients with primary pulmonary osteosarcoma internally. The area under the curve (AUC), sensitivity and specificity were 0.818, 0.848 and 0.800, respectively

We constructed a nomogram to predict the 1-year and 2-year OS, in which tumor size, microscopic findings of osteoid, surgical performance, and chemotherapy were included in the model (Fig. 2). The C-index of the prediction model was 0.771 (95% CI 0.691–0.851), and the AUC of the ROC curve was 0.818 (sensitivity, 0.848; specificity, 0.800; Fig. 3).

## Discussion

PPOS is one of the extraskeletal osteosarcomas that originated from the lung with an extremely low incidence and highly invasive potential [3]. Due to its extreme rarity, the clinical characteristics and prognosis of PPOS remain unclear. In this way, we collected the cases with PPOS from the SEER database and conducted a systematic literature review to explore the potential effective treatment and prognostic factors, which helped provide some suggestions for the management of PPOS. The median OS was eight months, and the 1-year OS rate was 30.8%. Besides surgical performance, tumor size and microscopic findings of osteoid were also identified as the independent prognostic factors, and a nomogram was successfully established to predict the prognosis of patients with PPOS with relatively high accuracy. Thus, our findings suggested that surgical resection might be considered for the primary treatment modality. To our knowledge, our research was the first study to demonstrate the clinical characteristics and prognosis of PPOS systematically and put forward a potential prediction model based on a relatively large scale.

PPOS was more common in males (65.3%), higher than the ratio reported before [3, 10]. The median age at diagnosis was 67 years, parallel with other studies [3]. Previously, age was significantly associated with survival [36], while different from our study, we did not find significant correlations between age and prognosis among patients with PPOS. Clinical symptoms might not be notable at an early stage of the disease, and chest pain was the most common as the tumor grew, which was in line with the previous reports [2, 10]. Metastases were only observed in 10 cases in our study, which was correlated with both OS and CSS in the univariable analysis. However, it was not an independent factor in the multivariable analysis. Therefore, the role of metastases in prognosis is required to be further studied because of the limited included cases with the metastasic disease. Our findings might remind us of the typical invasiveness and aggressiveness of PPOS regardless of metastasis.

The tumor size might refer to the local invasiveness, and tumor size > 7 cm indeed led to significantly poor survival, which was also an independent prognostic factor in the multivariable analysis. Previous studies were consistent with our findings that the size might be a critical indicator in determining the prognosis of patients with PPOS [12]. Currently, osteosarcoma was commonly evaluated by pathological grade, while pulmonary osteosarcoma was more recommended to be staged similar to the soft tissue sarcoma of thoracic visceral organs in the American Joint Committee On Cancer staging system, in which primary tumor was described with the depth of invasion and multifocal involvement instead of tumor size [37]. However, our study concluded that tumor size was a significant predictor of survival for patients with PPOS. Therefore, whether tumor size was supposed to be included in the staging system for PPOS required further investigation. Dyspnea was also a common clinical symptom due to the progression of disease and growth of tumor size [10, 18], and it was indicated that the median interval from the onset of clinical symptoms to diagnosis was three to four months, which also seemed to support for the invasiveness [23]. In this way, the growth rate might be a potential prognostic factor [25, 26]; however, it was not documented in detail and unavailable to be investigated properly.

The microscopic findings of the tumor were associated with the prognosis for PPOS. Those without osteoid were associated with a poor OS (HR = 2.11; *P* = 0.048). Notably, previous research found that there seemed to be some correlation between survival and different histological subtypes in patients with extraskeletal osteosarcoma [7]. In our study, the univariable analysis revealed that calcification was significantly correlated with survival. Generally speaking, calcification found in a mass on CT images was considered a benign sign, even though calcified findings were considered the specified characteristic of PPOS [2, 12]. Therefore, microscopic findings and radiographic manifestations might be significantly associated with the prognosis of PPOS, which required further validation. Furthermore, the microscopic findings could also be available for those who had not received surgical resection, as pathological examination could be conducted via transbronchial lung biopsy or needle biopsy. In addition, the presence of pleural effusion was associated with survival in univariable analysis but not identified as an independent prognostic factor in multivariable analysis. Due to the lack of details on whether these effusions were malignant, this variable was not included for further analysis.

Due to its rarity, only surgery was recommended as a potentially effective treatment for PPOS, and the role of chemotherapy and radiotherapy remains controversial even though chemoradiotherapy seemed to decrease the risk of distant metastasis and prolong the survival [2, 6, 7, 12, 13]. In our study, the Cox proportional hazard model showed that patients with surgery would have better OS (HR = 0.20, *P* < 0.001), which was in line with other studies [1, 2, 36]. The prognosis of patients with PPOS was extremely unsatisfactory, while those who received surgical resection might achieve nearly twice OS than those without surgery (ten versus five months). Thus, even though the potential invasiveness and aggressiveness were noted, surgery was still recommended. Furthermore, extensive radical resection was more recommended to remove the primary tumors and achieve a better local control [2, 7, 12]. Chapman reported a 33-year-old woman without a smoking history, who received surgery and adjuvant chemotherapy, survived 42 months postoperatively [29]. However, compared with en bloc resection or extensive surgery, inadequate resection was referred to as poor survival [22]. Therefore, it seemed essential to achieve a radical resection as possible when performing the first attempt of surgical resection. Radiotherapy was not effective, as shown in our findings, which was consistent with the previous studies [21, 30], while radiotherapy might be helpful in patients with distant metastasis [13, 33]. Moreover, even though the potential benefits of chemotherapy had been demonstrated in 266 patients with extraskeletal osteosarcoma [38], chemotherapy might not lead to a better OS in this study (*P* = 0.276).

What’s more, it is profitable to construct a prediction model to predict the prognosis of patients with rare diseases so as to help the clinicians make the right decision, and in this way, we constructed a nomogram, which could effectively assess the survival of those with PPOS (C-index, 0.771; AUC, 0.818). Unlike previous studies focusing on extraskeletal osteosarcoma [36], we merely studied the malignancy originating from the lung. To our knowledge, this is the first prediction model for PPOS, via which we might effectively predict the prognosis of patients with PPOS.

Our study had several limitations. As the data were collected from the reported cases and SEER database, the details of comorbidities, treatment contents and completeness of surgical resection were unavailable, which might lack persuasiveness to some extent. Secondly, due to the low incidence of PPOS, the included sample scale was not satisfactory, and there was no external cohort to further validate our conclusions. Moreover, including cases from the SEER database and case reports might create some data heterogeneity and introduce numerous biases. Therefore, our conclusions should be interpreted with caution and further multicenter studies are badly needed to better elucidate the clinical characteristics and prognosis of PPOS.

## Conclusion

PPOS is an uncommon malignancy of the lung with low incidence and rapid progression. The size of the tumor might be an important prognostic factor, and surgery could be a potentially effective treatment for patients with PPOS. A nomogram prediction model involving tumor size, pathological findings, and treatment modalities seemed to predict the prognosis of PPOS effectively. Further prospective multicenter studies, however, are required to confirm our conclusions.

## Supplementary Information

**Additional file 1. **Supplementary electronic tables.

## Data Availability

SEER* Stat (version 8.3.6) was employed for the first cohort, acquired in May 2020 at the SEER website (http://www.seer.cancer.gov).

## Abbreviations

AUC: Area under curve
CSS: Cancer-specific survival
CT: Computed tomography
CI: Confidential interval
C-index: Concordance index
HR: Hazard ratio
OS: Overall survival
PPOS: Primary pulmonary osteosarcoma
ROC: Receiver operating characteristic
SEER: Surveillance, Epidemiology, and End Result
