# Supplementary material for: Characteristics and prognosis of primary pulmonary osteosarcoma: a pooled analysis
Source: J Cardiothorac Surg. 2022 Sep 29;17:240. doi: 10.1186/s13019-022-02010-6 (PMC9520935; doi:10.1186/s13019-022-02010-6)
Supplement: Supplementary file 1 — Additional file 1. Supplementary electronic tables. [file 13019_2022_2010_MOESM1_ESM.docx]

**e-Table 1 Demographics, clinical characteristics of tumor, treatment and survival of patients with PPOS in SEER database**

| Year of diagnosis | Age | Sex | Location | Extent of disease | Size (cm) | Histology | Pathological Grade* | Treatment | Survival status | Survival (months) |
| --- | --- | --- | --- | --- | --- | --- | --- | --- | --- | --- |
| 1996 | 78 | Male | RL | Regional | 4 | ONOS | Undifferentiated | Surgery | Dead | 136 |
| 2001 | 61 | Male | RX | NA | NA | ONOS | NA | Surgery, chemotherapy | Dead | 19 |
| 2002 | 79 | Male | RL | Distant | 10 | ONOS | NA | Radiotherapy | Dead | 2 |
| 2003 | 73 | Male | LL | Localized | 6 | ONOS | NA | Surgery | Dead | 33 |
| 2004 | 83 | Male | RU | Regional | 4 | ONOS | Undifferentiated | Surgery | Dead | 3 |
| 2009 | 84 | Male | RU | Distant | 4.2 | ONOS | Poorly differentiated | Radiotherapy | Dead | 4 |
| 2010 | 94 | Female | RX | Distant | NA | ONOS | NA | None | Dead | 9 |
| 2011 | 56 | Female | RL | NA | NA | CO | Poorly differentiated | Surgery | Dead | 0 |
| 2012 | 67 | Female | LX | Localized | 16.5 | FO | NA | Chemotherapy | Dead | 3 |
| 2013 | 73 | Male | LX | Distant | 1.8 | ONOS | Undifferentiated | None | Dead | 0 |
| 2015 | 84 | Male | LU | Localized | 8 | ONOS | Undifferentiated | Surgery | Dead | 10 |

*Pathological Grade: the histological grading and differentiation of tumor based on ICD-O-3 Morphology Codes.

**Abbreviations:** PPOS, primary pulmonary osteosarcoma; RL, right lower lobe; RU, right upper lobe; RX, right lung(the location of the pulmonary lobe was not specified); LL, left lower lobe; LU, left upper lobe; LX, left lung(the location of the pulmonary lobe was not specified); NA, not available; ONOS, osteosarcoma(not otherwise specified); CO, chondroblastic osteosarcoma; FO, fibroblastic osteosarcoma.

**e-Table 2 Demographics, clinical manifestations, imaging manifestations, procedures for definite diagnosis and histology of microscopy findings of patients with PPOS in case series**

| No. | Case report | Age | Sex | Clinical manifestations | Imaging manifestations | Procedures for definite diagnosis | Histology of microscopy findings |
| --- | --- | --- | --- | --- | --- | --- | --- |
| 1 | Greenspan 1933 | 35 | F | Chest pain, dizziness, nausea, fever, sputum, hoarseness | PE | Autopsy | Osteoid, CHON |
| 2 | Nosanchuk 1969 | 66 | M | Weakness, dyspnea, chest pain, sputum, hemoptysis, fever | PE, CPM* | Autopsy | OSTEO, CHON |
| 3 | Reingold 1971 | 62 | M | Fever, sputum, asthma, pneumonia | NA | Autopsy | OSTEO, Osteoid, CHON |
| 4 | Reingold 1971 | 56 | F | Chest pain, chills, fever | NA | Surgery | OSTEO, Osteoid, CHON |
| 5 | Nascimento 1982 | 74 | F | Asymptomatic | NA | Surgery | OSTEO |
| 6 | Nascimento 1982 | 72 | M | Asymptomatic | NA | Surgery | OSTEO |
| 7 | Colby 1989 | 81 | M | Dyspnea | NA | NA | NA |
| 8 | Colby 1989 | 51 | M | Cough | NA | NA | NA |
| 9 | Colby 1989 | 77 | F | Pneumonia | NA | NA | NA |
| 10 | Loose 1990 | 54 | M | Chest pain, paresthesia | CPM | Surgery | OSTEO, Osteoid |
| 11 | Loose 1990 | 45 | F | Chest pain | NA | Surgery | NA |
| 12 | Kimura 1990 | 44 | F | Cough, dyspnea | CPM, IUBS | TBLB | OSTEO |
| 13 | Petersen 1990 | 70 | M | Asymptomatic | CPM, IUBS | Surgery | OSTEO, Osteoid, CHON |
| 14 | Stark 1990 | 59 | M | Asymptomatic | PE, CPM | Surgery | OSTEO |
| 15 | Connolly 1991 | 93 | M | Asymptomatic | PE, CPM, IUBS | Needle biopsy | OSTEO, Osteoid |
| 16 | Bhalla 1992 | 58 | M | Fever, cough | PE, CPM | Autopsy | OSTEO, CHON |
| 17 | Miller 1993 | 72 | M | NA | NA | TBLB | NA |
| 18 | Fujii 1998 | 66 | F | Dyspnea | NA | Autopsy | OSTEO, Osteoid |
| 19 | Wagner 1999 | 48 | M | Chest pain | PE, CPM | Fine-needle biopsy | OSTEO, Osteoid, CHON |
| 20 | Sievert 2000 | 56 | M | Tingling in left fingertips | NA | Surgery | Osteoid |
| 21 | Chapman 2001 | 33 | F | Cough, chest pain | IUBS | Surgery | OSTEO, Osteoid |
| 22 | Magishi 2004 | 74 | F | Asymptomatic | NA | Surgery | OSTEO, Osteoid, CHON |
| 23 | Tsunezuka 2004 | 58 | F | Dyspnea, chest pain | NA | Surgery | Osteoid, CHON |
| 24 | Kadowaki 2005 | 72 | M | Dyspnea, chest pain | PE, CPM, IUBS | Needle biopsy | Osteoid |
| 25 | Kadowaki 2005 | 77 | M | Back pain, limb edema, hemosputum | CPM, IUBS | Autopsy | OSTEO, Osteoid |
| 26 | Langer 2006 | 65 | M | COPD | CPM | Fine-needle biopsy | Osteoid |
| 27 | Yamazaki 2006 | 73 | M | Cough, hemosputum | NA | TBLB | OSTEO, Osteoid |
| 28 | Niimi 2008 | 72 | M | Dyspnea | PE, CPM, IUBS | Needle biopsy | OSTEO, Osteoid, CHON |
| 29 | Matono 2008 | 74 | M | Cough, dyspnea | PE, CPM | Surgery | OSTEO, Osteoid, CHON |
| 30 | Elias 2010 | 58 | F | Asymptomatic | CPM | Surgery | OSTEO, CHON |
| 31 | Lee 2010 | 43 | F | NA | NA | NA | OSTEO |
| 32 | Wajstaub 2011 | 77 | M | Asymptomatic | NA | Needle biopsy | Osteoid |
| 33 | Shenjere 2011 | 66 | M | Dyspnea, chest pain | NA | TBLB | Osteoid, CHON |
| 34 | Shenjere 2011 | 79 | M | Dyspnea | NA | Autopsy | Osteoid, CHON |
| 35 | Shenjere 2011 | 56 | F | Cough | NA | Surgery | Osteoid, CHON |
| 36 | Shenjere 2011 | 58 | F | Chest pain，cough | NA | Surgery | Osteoid, CHON |
| 37 | Gu 2011 | 58 | M | Chest pain | CPM, IUBS | Surgery | OSTEO, CHON |
| 38 | Makoto 2014 | 75 | M | Asymptomatic | CPM, IUBS | Fine-needle biopsy | Osteoid |

*CPM: calcification found in pulmonary mass on chest roentgenograms.

**Abbreviations:** PPOS, primary pulmonary osteosarcoma; F, female; M, male; NA, not available; PE, Pleural effusion; CPM, calcification found in pulmonary mass on computerized tomography; IUBS, markedly intensive uptake on ^99^mTc- methylene bisphosphonate bone scintigraphy; TBLB, transbronchial lung biopsy; CHON, chondrocyte, chondroblast or chondroid; OSTEO, osteocyte, osteoblast or osteoclast.

**e-Table 3** **Clinical characteristics of tumor, treatment and prognosis of patients with PPOS in case series**

| No. | Case report | Number of RIST | Location | Size  (cm) | Metastasis | Treatment | Survival status | Survival (months) |
| --- | --- | --- | --- | --- | --- | --- | --- | --- |
| 1 | Greenspan 1933 | 1 | LU, Central | 7 | Metastasis | None | DOD | 11* |
| 2 | Nosanchuk 1969 | 3 | LW, Central | >10 | Metastasis | None | DOD | 5* |
| 3 | Reingold 1971 | 1 | RM, Peripheral | 6 | No metastasis | Chemotherapy | DOD | 8* |
| 4 | Reingold 1971 | 1 | LU, Peripheral | 7.5 | No metastasis | Surgery | ALF | 14 |
| 5 | Nascimento 1982 | NA | RM, NA | 4 | No metastasis | Surgery | DUC | 8 |
| 6 | Nascimento 1982 | NA | RU, NA | 5.5 | No metastasis | Surgery | DUC | 10 |
| 7 | Colby 1989 | NA | RW, NA | >10 | No metastasis | Surgery | DOC | NA |
| 8 | Colby 1989 | NA | LL, NA | >10 | No metastasis | Surgery | ALF | 6 |
| 9 | Colby 1989 | NA | RM, NA | <5 | No metastasis | Surgery | DOD | 6 |
| 10 | Loose 1990 | 1 | LU, Peripheral | 10 | No metastasis | Surgery, Chemotherapy, Radiotherapy | ALF | 7 |
| 11 | Loose 1990 | 2 | LL, Peripheral | 5.5 | No metastasis | Surgery, Chemotherapy | ALF | 2 |
| 12 | Kimura 1990 | 1 | RW, Central | 12 | No metastasis | Surgery, Radiotherapy | DOD | 7 |
| 13 | Petersen 1990 | 1 | LL, Peripheral | 6 | No metastasis | Surgery, Radiotherapy | ALF | 6 |
| 14 | Stark 1990 | 1 | LL, Peripheral | 11 | No metastasis | Surgery | NA | NA |
| 15 | Connolly 1991 | 1 | LU, Peripheral | 4 | No metastasis | None | DOD | 12 |
| 16 | Bhalla 1992 | 1 | LU, Central | 18 | No metastasis | None | DOD | 1* |
| 17 | Miller 1993 | 1 | RX, NA | NA | Metastasis | Chemotherapy, Radiotherapy | DOD | 12 |
| 18 | Hirofumi 1998 | 1 | RX(PA), Central | 5 | Metastasis | None | DOD | 13* |
| 19 | Wagner 1999 | 1 | RL, Peripheral | 5.1 | No metastasis | None | DOD | 1 |
| 20 | Sievert 2000 | 2 | LU, Peripheral | 4 | No metastasis | Surgery | ALF | 12 |
| 21 | Chapman 2001 | 1 | LU, Central | 5.5 | No metastasis | Surgery, Chemotherapy, Radiotherapy | ALF | 42 |
| 22 | Magishi 2004 | 1 | LU, Central | 5.7 | No metastasis | Surgery | DOD | 11 |
| 23 | Tsunezuka 2004 | 1 | RU(PA), Central | NA | No metastasis | Surgery | ALF | 24 |
| 24 | Kadowaki 2005 | 1 | LL, Central | 9 | Metastasis | None | DOD | 3 |
| 25 | Kadowaki 2005 | 1 | RL, Peripheral | 11 | No metastasis | None | DOD | 3* |
| 26 | Florian 2006 | 2 | RL, Peripheral | 5.5 | No metastasis | Surgery, Chemotherapy, Radiotherapy | DOD | 9 |
| 27 | Koji 2006 | 1 | LU, Peripheral | 7.2 | No metastasis | Surgery | DOD | 7 |
| 28 | Niimi 2008 | 1 | LL, Central | 9.5 | No metastasis | None | DOC | 5 |
| 29 | Matono 2008 | 1 | LL, Peripheral | 11.3 | No metastasis | Surgery | NA | NA |
| 30 | Elias 2010 | 1 | LU, Peripheral | 2.5 | No metastasis | Surgery, Chemotherapy, Radiotherapy | ALF | 6 |
| 31 | Lee 2010 | 1 | NA, NA | 7 | No metastasis | Surgery | DOD | 15.6 |
| 32 | Sandra 2011 | 2 | RL, Central | 5 | No metastasis | Surgery, Radiotherapy | DOD | 25 |
| 33 | Shenjere 2011 | 1 | LU, Central | 6 | No metastasis | Surgery | ALF | 12 |
| 34 | Shenjere 2011 | 1 | LU, Peripheral | 11 | Metastasis | Radiotherapy | DOD | 5* |
| 35 | Shenjere 2011 | 1 | RL, Peripheral | 3.2 | No metastasis | Surgery | NA | NA |
| 36 | Shenjere 2011 | 1 | LL, NA | 25 | No metastasis | Surgery | NA | NA |
| 37 | Gu 2011 | 3 | RU, RM, RL, Peripheral | 8 | No metastasis | Surgery, Chemotherapy | NA | NA |
| 38 | Makoto 2014 | 2 | LU, Peripheral | 4 | No metastasis | Surgery | DOD | 12 |

*Survival(months): The survival time with asterisk referred to the cases diagnosed by autopsy, indicating that the duration was calculated from the onset of disease to death.

**Abbreviations:** PPOS, primary pulmonary osteosarcoma; RIST, recorded in situ tumors; NA, not available; LU, left upper lobe; LL, left lower lobe; LW, left side of the whole lung; RU, right upper lobe; RM, right middle lobe; RL, right lower lobe; RW, right whole lung; RX, right lung(the location of the pulmonary lobe was not specified); PA, close to the pulmonary artery; DOD, dead of disease; ALF, alive at the last follow-up; DUC, died of unknown reasons; DOC, died of other causes.
